# Menu-engineering in restaurants - adapting portion sizes on plates to enhance vegetable consumption: a real-life experiment

**DOI:** 10.1186/s12966-017-0496-9

**Published:** 2017-04-19

**Authors:** Machiel J. Reinders, Marlijn Huitink, S. Coosje Dijkstra, Anna J. Maaskant, Joris Heijnen

**Affiliations:** 1 Wageningen University and Research, Wageningen Economic Research, P.O. Box 29703, 2502 LS The Hague, The Netherlands; 2 Vrije Universiteit Amsterdam, de Boelelaan 1085, 1081 HV Amsterdam, The Netherlands; 3 Wageningen University and Research, Wageningen Food and Biobased Research, P.O. Box 17, 6700 AA Wageningen, The Netherlands; 4 Variatie in de Keuken, Stichting Variatie op de Kaart, H.J.E. Wenckebachweg 47A, 1096 AK Amsterdam, The Netherlands

**Keywords:** Vegetables, Restaurant, Menu, Intake, Portion size, Meat, Diet

## Abstract

**Background:**

The aim of this research was to investigate whether increased portion sizes of vegetables and decreased portion sizes of meat on main dishes increased the amount of vegetables consumed in a real-life restaurant setting without affecting customer satisfaction. The participants were unaware of the experiment.

**Methods:**

A cross-over design was used in which three restaurants were randomly assigned to a sequence of an intervention and control condition. In the intervention period, the vegetable portion sizes on the plates of main dishes were doubled (150 g of vegetables instead of 75 g) and the portion sizes of meat on the plates were reduced by an average of 12.5%. In the control period, the portion sizes of the main dishes were maintained as usual. In total, 1006 observations and questionnaires were included.

**Results:**

Vegetable consumption from plates was significantly higher during the intervention period (*M* = 115.5 g) than during the control period (*M* = 61.7 g). Similarly, total vegetable consumption (including side dishes) was significantly higher during the intervention period (*M* = 178.0 g) than during the control period (*M* = 137.0 g). Conversely, meat consumption was significantly lower during the intervention period (*M* = 183.1 g) than during the control period (*M* = 211.1 g). Satisfaction with the restaurant visit did not differ between the intervention period (*M* = 1.27) and control period (*M* = 1.35). Satisfaction with the main dish was significantly lower during the intervention period (*M* = 1.25) than during the control period (*M* = 1.38), although in both cases, the scores indicated that participants remained (very) satisfied with their main dish.

**Conclusions:**

This study showed that increasing vegetable portions in combination with decreasing meat portions (unknowingly to the consumer) increased the amount of vegetables consumed and decreased the amount of meat consumed. Furthermore, despite the changes in portion sizes, participants remained satisfied with their restaurant visit and main dish. The findings of this study suggest that modifying portion size in restaurants is an effective tool for stimulating vegetable consumption and consequently healthy and sustainable diets.

## Background

Healthy eating and drinking, alongside adequate exercise, is one of the most important ways of growing old while maintaining health and vitality. In this respect, a large body of evidence shows that a high vegetable intake helps promote health and prevent diet-related chronic disease [1, 2]. Despite the considerable evidence that eating vegetables has various health benefits, individuals consume less than the recommended amount in most Western countries [3].

This lower intake coincides with an increase in the number of meals consumed away from home, in restaurants or at fast food outlets [4, 5]. Increased consumption outside of the home is associated with increased intake of large portions of unhealthy, high calorie foods with fewer vegetables, which may subsequently contribute to the increasing prevalence of overweight and obesity in Western countries [6, 7]. Furthermore, the volume of meat consumed in restaurants is high [8]. This high level of consumption has presented challenges to food sustainability, including loss of arable land and biodiversity [9] and other animal welfare [10], public health [11, 12] and climate change [13] effects. Therefore, restaurants could play an important role in mitigating the increase in overweight and obesity and could improve diet quality by offering healthier and more sustainable food choices (i.e., more vegetables, less meat) on their menus [14]. In addition, it could be argued that dietary change interventions can have the greatest impact when they are conducted in “limited access” sites, i.e., restaurants and canteens, instead of grocery stores, as it is easier in these settings to sway customers towards making healthier choices [15].

One way restaurants can promote healthier food choices is by modifying portion sizes [16]. When people are served larger portions, they eat more than when they are offered smaller portions [17]. The results of a field study by Diliberti et al. showed that portion size significantly affected how much food was consumed, while a satisfaction survey showed no difference in the ratings of the appropriateness of the two different portions with higher and lower portion sizes [18]. Others found that reducing the portion sizes of items in university restaurants resulted in reduced dietary intake and also in reduced plate waste [19]. An experiment by Wansink et al. showed that participants who were unknowingly eating from self-refilling bowls ate more soup than those eating from normal soup bowls [20]. Despite consuming 73% more, the participants eating from self-refilling bowls did not perceive themselves to be more sated, nor did they believe that they had consumed more than those eating from normal bowls. The authors argued that the amount of food on a plate or bowl is associated with the amount of intake because it influences consumption norms and expectations. Increasing the size of portions or the variety of healthy products with a low energy density, such as vegetables, has also been shown to be effective in decreasing overall energy intake and promoting healthy eating. Rolls et al. investigated the effects of varying the portion size and energy density of a first course salad on overall ad libitum lunch intake [21]. Compared to having no first course, consuming a salad with the lowest energy density reduced meal intake by 7% (268 kJ) for the small portion and 12% (448 kJ) for the large portion. Although subjects consumed significantly less energy at the meal with the large low-energy-dense salad, they felt similarly full as when they consumed meals with other large salads. Others showed that having a choice in the variety of vegetables during a lunch buffet enhanced healthier food choices [22]. Giving participants one additional vegetable to choose from increased the total as well as the relative energy provided by vegetables. In contrast, the relative energy derived from non-vegetables (e.g., pasta and chicken) significantly decreased. Furthermore, consuming a low-energy-dense broth-based soup as a first course has also been found to reduce overall meal intake [23].

This experimental evidence suggests that changing both portion sizes and the energy density of foods or menus is potentially effective in maintaining satiety, reducing energy intake and increasing vegetable intake in the short term. Therefore, the aim of this research is to investigate whether increased portion sizes of vegetables and decreased portion sizes of meat^1^ in a main dish increase the amount of vegetables consumed in a real-life restaurant setting without affecting customer’s satisfaction. Furthermore, we examine to what extent the quantity of vegetable and meat consumption is associated with demographic, lifestyle and nutrition-related characteristics.

We hypothesized that *increasing the portion size of healthy food products (*i.e.*, vegetables) leads to a higher consumption of these healthy food products and that decreasing the portion size of meat will lead to a lower consumption of meat products*. Furthermore, we hypothesized that the adaption of portion sizes *will not lead to a decrease in customer satisfaction*. Because eating behaviors and also satiety differ between distinct subgroups in the population, the characteristics of the restaurants’ visitors are included in this study. By considering this information, we aim to provide insight into the potential effect of our intervention.

## Methods

### Context and design

The study was conducted within the public-private partnership “Meer groente & fruit voor iedereen” (More vegetables and fruit for everyone), which was sponsored by the Dutch Ministry of Economic Affairs and the Fresh Produce Centre. The study started in May 2015 and was implemented in three restaurant locations of a major restaurant chain in medium-sized cities in the Netherlands. The participating restaurants were selected by convenience sampling, and to obtain a representative sample, restaurants were distributed throughout different parts of the Netherlands. Furthermore, the particular restaurant chain was selected because it receives guests from all levels of the population, further enhancing the representativeness of the data. To recruit restaurants, an email was sent that explained the rationale of the study and included an invitation to sign up to participate in the study. After this email, restaurants were called to determine their interest in participating. After agreeing, the researcher visited each restaurant to meet and discuss the intervention. A few weeks before the intervention started, the restaurants had to sign an agreement in which they accepted the study design. During the intervention, restaurants were phoned weekly by the researcher to discuss the progress of the intervention.

We used a cross-over design in which each restaurant was randomly assigned to a sequence of an intervention or control condition. Location one and three started with a 6-week control period followed by a 6-week intervention period. Location two started in the opposite direction. Measurements took place on Saturday evenings between 6 pm and 10 pm (*n* = 12 per location).

### Sample size

Approximately 400 guests visited the restaurants each evening. However, only guests who ordered one of the relevant menu items were included in the study. Moreover, due to the maximum capacity of the research assistants in the kitchen, tables with more than twelve plates were excluded from our study. This resulted in a total of 2080 unique plates that were measured. After removing plates that were measured but were not included in the study (e.g., vegetarian meals, child menus and special offerings (e.g., today’s menu)), 1735 observations remained. After deleting observations without a completed questionnaire, 1193 observations were left. Of these, 187 questionnaires were excluded due to missing data, resulting in a total of 1006 observations and questionnaires included in this study (control period *N* = 536, intervention period *N* = 470).

### Intervention description

During the intervention period, the portion sizes of vegetables on the plates of main dishes were doubled (150 g of vegetables instead of 75 g) and the portion sizes of meat were reduced by an average of 12.5%. In the control period, the portion sizes of the main dishes remained as usual.

During the intervention period, the following provisions were applied in all locations:

1. Plate:
  - A mix of vegetables served per plate, prepared in the same way with the same quantity of vegetables for every plate (75 g during control weeks and 150 g during intervention weeks).
  - The same starch product (potato gratin) provided per plate, with approximately the same quantity for every plate (same for control and intervention weeks).
2. Side dish:
  - The same type of vegetables served in every bowl, prepared in the same way, with approximately the same quantity of vegetables for each bowl (same during control and intervention period). The type of vegetables in the side dish differed from the vegetable mix on the plate.
  - The same starch product (one fries and one baked potato) provided for every bowl at approximately the same quantity (same during control and intervention weeks).

### Procedures

Plate measurements were conducted by two to three trained research assistants per location on Saturday evenings between 6 pm and 10 pm. First, there was a check at the beginning of the evening. At least five samples of each component of the plates (meat or fish, vegetables and potato gratin) and side dishes (vegetables, fries and baked potatoes) that were included in the study were measured on a calibrated scale to measure the weight before and after preparation of the component. Based on these measurements, an average weight of each component was calculated and was used to obtain the average serving portion for that specific main dish or side dish. This check was performed three times during the intervention and three times during the control period. To serve the correct amount of vegetables (75 g or 150 g) on the plates of main dishes, customized spoons were used that corresponded to the correct amount. Furthermore, the chefs practiced serving the right amount of vegetables at least five times per evening, and the amount was also checked by a calibrated scale.

After guests consumed their main dishes, the remaining amount of vegetables, meat, and potato gratin as well as the food left over from the side dishes (vegetables, fries, baked potatoes) were weighed when they returned to the kitchen. Based on these measurements, vegetable consumption was calculated by subtracting the weight of the remaining vegetables on the plates returned to the kitchen from the average amount of vegetables served during the control (75 g) and intervention (150 g) period. Meat consumption was calculated in the same manner by subtracting the weight of the remaining meat on the plates returned to the kitchen from the average amount of meat served in the control and intervention period. The research assistants attempted to measure as many of the plates returned to the kitchen as possible.

### Questionnaire

After the waiter/waitress collected the plates, the diners were asked to participate in a study about the satisfaction and consumption behavior of restaurant customers conducted in cooperation with Wageningen University & Research and Stichting Variatie in de Keuken. If they agreed, they were asked to complete a questionnaire, which took on average five to ten minutes. The participants remained unaware of the experiment. Unfortunately, we did not monitor the number of participants who refused participation, but according to the waiters/waitresses and research assistants, this number was very low. Reasons for not participating included that customers had already participated, had no time, or felt no need to participate.

In the questionnaire, participants were first asked to indicate their menu choice (main dish). After that, they indicated their reasons for visiting the restaurant (i.e., business or leisure), the frequency of visiting the particular restaurant (ranging from ‘4 times a month or more’ to ‘this is the first time’) and how often they dine out in general (ranging from ‘4 times a month or more’ to ‘1–2 times a year or less’). The participants then had to rate their satisfaction with the consumed main dish on a 5-point Likert scale ranging from very dissatisfied to very satisfied. Similarly, they indicated their satisfaction with their visit to the restaurant. This item was also rated on a 5-point Likert scale ranging from very dissatisfied to very satisfied. In addition, participants were asked to provide their opinion about the amount of vegetables and meat that was served with their main dish. These two items were rated on a 5-point Likert scale ranging from too much to too little. Additionally, participants assessed the importance of vegetables when dining, with responses ranging from very unimportant to very important. Each response for the 5-point Likert scale questions was scored from −2 to +2. Furthermore, respondents were asked to complete statements on a 7-point Likert scale from totally disagree to totally agree about food involvement [24], liking of vegetables [24], and subjective knowledge about vegetables [25]. Each response on the 7-point Likert scale was scored from −3 to +3. Finally, demographic questions were asked, including gender, age, level of education and having children.

### Data analyses

We used descriptive statistics to summarize the participants' characteristics. Continuous variables were presented as the means and standard deviations, whereas categorical variables were presented as relative frequencies. Independent samples *t*-tests were used to test whether the means differed significantly between the intervention and control groups.

The dependent variables were vegetable consumption in grams per plate, vegetable consumption in total grams (plate + side dish), meat/fish consumption and satisfaction with the restaurant visit.

One-way ANOVA revealed significantly differences between the three locations regarding the dependent variables, except for vegetable consumption in total grams (*F* = 1.24, *p* > 0.05). Accordingly, we decided to control for location and incorporated it as an extra factor in the analyses. Subsequently, MANOVAs were conducted with vegetable consumption from the plate, total vegetable consumption (including side dishes), total meat consumption, satisfaction with the main dish and satisfaction with the restaurant as the dependent variables and the condition (intervention versus control) and location as factors.

For subgroups that could be distinguished based on categorical variables (i.e., gender, having children, reason for visit), independent samples t-tests were used to test whether these subgroups differed in the amount of vegetables and meat consumed. For participant characteristics that were measured by continuous variables (i.e., age, frequency of visit, frequency of dining out, involvement with food, liking of vegetables), correlational analyses were used to test whether there were positive or negative associations with vegetable and meat consumption.

## Results

In total, we included 1006 participants: 536 in the control condition and 470 in the intervention condition. In our study sample, 54% were women and 46% were men, and the age of the respondents ranged from 9 to 88 years (*M* = 48.6, SD = 17.5). With regard to education level, approximately 37% of the respondents reported having a high education level (i.e., college or university), 28% a medium education level (i.e., vocational education) and 35% a low education level (i.e., primary education or secondary education). Other baseline characteristics of the sample are described in Table 1. In addition, Table 2 shows the means and standard deviations of the questionnaire items for the total study population as well as for the control and intervention period. With the exception of the dependent variables in this study, the independent samples *t*-tests revealed no significant differences between the control period and the intervention period for the measured items. One exception was the importance of vegetables when dining out; scores for this variable were significantly lower during the intervention period than the control period, although this question was not related to the intervention.

**Table 1:** Characteristics of study population

	Total study population (*N* = 1006)	Intervention period (*N* = 536)	Control period (*N* = 470)
Age (mean)	48.6 years	47.9 years	49.3 years
Gender:			
Male	46.0%	44.1%	47.7%
Female	54.0%	55.9%	52.3%
Education:			
High level (i.e., college or university)	36.6%	35.8%	37.3%
Medium level (i.e., vocational education)	28.4%	28.4%	28.3%
Low level (i.e., primary education or secondary education	35.0%	35.8%	34.4%
Children:			
Yes	68.8%	69.8%	67.9%
No	31.2%	30.2%	32.1%
Reason restaurant visit:			
Private (leisure) reasons	97.3%	96.8%	97.8%
Business reasons	2.7%	3.2%	2.2%
Frequency restaurant visit:			
First time	42.9%	43.2%	42.6%
1–2 times a year or less	25.6%	25.2%	26.0%
3–6 times a year	20.2%	22.0%	18.7%
7 times a year or more	11.3%	9.6%	12.7%
Frequency going out for dinner:			
Once a month or more	33.8%	35.1%	32.6%
10–12 times a year	20.9%	18.8%	22.7%
7–9 times a year	15.3%	15.2%	15.4%
3–6 times a year	23.9%	26.1%	21.9%
1–2 times a year or less	6.2%	4.7%	7.5%

**Table 2:** Means (SD) of questionnaire items for total study population and control and intervention period

	Total study population (*N* = 1006)	Control period (*N* = 470)	Intervention period (*N* = 536)	Difference test (*t*-value)
Satisfaction with meal [−2 = very dissatisfied; 2 = very satisfied]	1.35 (.62)	1.41 (.59)	1.29 (.64)	2.97**
Satisfaction with restaurant visit [−2 = very dissatisfied; 2 = very satisfied]	1.34 (.55)	1.37 (.55)	1.31 (.56)	1.74
Evaluation amount of vegetables [−2 = too little; 2 = too much]	.13 (.70)	−.07 (.61)	.37 (.73)	−10.18***
Evaluation amount of meat/ fish [−2 = too little; 2 = too much]	.15 (.54)	.19 (.53)	.11 (.54)	2.41*
Importance of vegetables when dining [−2 = very unimportant; 2 = very important]	.93 (.85)	.98 (.81)	.86 (.89)	2.17*
I highly value a tasteful meal.^a^	2.56 (1.01)	2.56 (1.07)	2.57 (.94)	−.16
I am very involved with my food choices.^a^	1.78 (1.31)	1.75 (1.32)	1.81 (1.29)	−.73
I have been eating vegetables since I was a child.^a^	2.22 (1.30)	2.27 (1.29)	2.17 (1.32)	1.29
I am very particular about the vegetables I will eat.^a^	.91 (1.82)	.90 (1.80)	.92 (1.84)	−.14
I like vegetables.^a^	1.65 (1.50)	1.71 (1.48)	1.59 (1.53)	1.28
I would like to eat more vegetables.^a^	.50 (1.76)	.57 (1.74)	.42 (1.77)	1.35
I know how much vegetables I have to eat during the day.^a^	1.55 (1.75)	1.56 (1.72)	1.54 (1.79)	.16
I find it difficult to assess whether I eat enough vegetables.^a^	−.49 (2.03)	−.56 (2.03)	−.40 (2.04)	−1.25

^a^Answering scales range from −3 = totally disagree to +3 = totally agree; *** *p* < .001; **p < .01; *p < .05

### Vegetable and meat consumption

The results of MANOVAs revealed that vegetable consumption from plates was significantly higher during the intervention period (*M* = 115.5, SE = 1.82) than during the control period (*M* = 61.7, SE = 1.65 *F*(1, 993) = 478.92, *p* < .001) (Table 3). The difference equaled an increase in vegetable consumption of approximately 87%. Similarly, total vegetable consumption (including side dishes) was significantly higher during the intervention period (*M* = 178.0, SE = 2.92) than during the control period (*M* = 137.0; SE = 2.65 *F*(1, 993) = 108.2, *p* < .001). Note that the total increase in vegetable consumption (30%) was less than the increase in vegetable consumption from the plates because the participants ate fewer vegetables from the served side dishes in the intervention period. Furthermore, meat consumption was significantly lower during the intervention period (*M* = 183.1, SE = 2.52) than during the control period (*M* = 211.1; SE = 2.29 *F*(1, 993) = 67.75, *p* < .001). This difference equaled a decrease of approximately 13%, which corresponds with the fact that the amount of meat served in the intervention period was on average 12.5% lower. These results support the hypothesis that increasing the portion size of vegetables leads to a higher consumption of these products and that decreasing the portion size of meat will lead to a lower consumption of meat products.

**Table 3:** Estimated marginal means (SE) for vegetable and meat/fish consumption in grams for all three locations

		Vegetable consumption in grams (plate)	Vegetable consumption in grams (total, incl. side dishes)	Meat/fish consumption in grams
Period				
Control (*n* = 530)		61.7 (1.65)^a^	137.0 (2.65)^a^	211.1 (2.29)^a^
Intervention (*n* = 469)		115.5 (1.82)^b^	178.0 (2.92)^b^	183.1 (2.52)^b^
Location				
Restaurant 1 (*n* = 129)		90.0 (2.89)^a^	166.2 (4.63)^a^	203.7 (4.01)^a^
Restaurant 2 (*n* = 324)		87.3 (1.81)^a^	159.3 (2.90)^a^	197.1 (2.51)^b^
Restaurant 3 (*n* = 546)		88.5 (1.40)^a^	147.0 (2.24)^b^	190.6 (1.94)^b^
Main effect intervention	*F* (df1,df2) Partial *η* ^2^	478.92*** (1, 993) .325	108.2*** (1, 993) .098	67.75*** (1, 993) .064
Main effect location	*F* (df1,df2) Partial *η* ^2^	.35 (2, 993) .001	9.97*** (2, 993) .020	5.23** (2, 993) .010
Main effects intervention*location	*F* (df1,df2) Partial *η* ^2^	7.55** (2, 993) .015	2.87 (2, 993) .006	9.31*** (2, 993) .018

Reported means are amount in grams (standard errors are reported in brackets); Means with a different superscript ^a, b^ indicate a significant difference (*p* < .05) (means are compared two at a time); *** *p* < .001; **p < .01; *p < .05

The results also showed significant interaction effects between the intervention and the location of the restaurants on vegetable consumption from plates (*F*(2, 993) = 7.55, *p* < .01) and on meat consumption *F*(2, 993) = 9.31, *p* < .001). Inspection of the means indicated that the differences in vegetable consumption from plates between the intervention and control conditions were significantly larger in location 1 than location 2. Similarly, we found that the differences in meat consumption between the intervention and control conditions were significantly larger in location 1 than in location 3.

### Evaluation and satisfaction

The results of MANOVAs revealed that the participants evaluated the amount of vegetables to be significantly higher in the intervention period (*M* = .32, SE = .04) than in the control period (*M* = −.12; SE = .03 *F*(1, 1000) = 73.30, *p* < .01) (Table 4). However, the evaluation of both conditions was slightly above or below the midpoint of the scale (not too much, not too little), indicating that the amount of vegetables was still acceptable to the participants. In addition, participants evaluated the amount of meat to be significantly lower in the intervention condition (*M* = .08; SE = .03) than in the control condition (*M* = .18; SE = .03 *F*(1, 1000) = 6.26, *p* < .05). Again, these scores indicated that for both conditions, the amount of meat was still acceptable (not too much, not too little).

**Table 4:** Estimated marginal means (SE) for evaluation amount of vegetables and meat/fish and satisfaction for all locations

		Evaluation amount of vegetables [−2 = too little; 2 = too much]	Evaluation amount of meat or fish [−2 = too little; 2 = too much]	Satisfaction with meal [−2 = very dissatisfied; 2 = very satisfied]	Satisfaction with restaurant visit [−2 = very dissatisfied; 2 = very satisfied]
Period					
Control (*n* = 530)		−.12 (.034)^a^	.18 (.027)^a^	1.38 (.031)^a^	1.35 (.028)^a^
Intervention (*n* = 469)		.32 (.037)^b^	.08 (.030)^b^	1.25 (.034)^b^	1.27 (.031)^a^
Location					
Restaurant 1 (*n* = 129)		−.01 (.059)^a^	.06 (.048)^a^	1.26 (.054)^a^	1.23 (.049)^a^
Restaurant 2 (*n* = 324)		.12 (.037)^b^	.14 (.030)^a^	1.25 (.034)^a^	1.29 (.030)^a^
Restaurant 3 (*n* = 546)		.19 (.029)^b^	.18 (.023)^a^	1.43 (.026)^b^	1.40 (.024)^b^
Main effect Intervention	*F* (df1,df2) Partial *η* ^2^	73.30*** (1, 1000) .068	6.26* (1, 1000) .006	7.20** (1, 1000) .007	3.65 (1, 1000) .004
Main effect Location	*F* (df1,df2) Partial *η* ^2^	5.16** (2, 1000) .010	2.36 (2, 1000) .005	10.22*** (2, 1000) .020	6.95** (2, 1000) .014
Main effects Intervention*Location	*F* (df1,df2) Partial *η* ^2^	1.83 (2, 1000) .004	.43 (2, 1000) .001	.94 (2, 1000) .002	1.27 (2, 1000) .003

Answer scales ranged from −2 to 2 (standard errors are reported in brackets); Means with a different superscript ^a, b^ indicate a significant difference (*p* < .05) (means are compared two at a time); *** *p* < .001; **p < .01; *p < .05

Satisfaction with the main dish was significantly lower during the intervention period (*M* = 1.25, SE = 0.03) than during the control period (*M* = 1.38; SE = 0.03 *F*(1, 1000) = 7.20, *p* < .01). However, during the intervention condition, satisfaction with the main dish was above the midpoint of the answer scale (0), indicating that the participants were still (very) satisfied with their meal. Furthermore, satisfaction with the restaurant visit did not differ between the intervention period (*M* = 1.27, SE = .31) and the control period (*M* = 1.35; SE = .03 *F*(1, 1000) = 3.65, *p* > .05). In both conditions, these scores indicated that the participants were (very) satisfied with the restaurant. Taken together, partial support was found for the hypothesis that increasing the portion size of vegetables and decreasing the portion size of meat would not lead to a decrease in customer satisfaction.

### Sub-sample analyses

We further explored whether the amount of vegetables and meat consumed differed for different subsamples (Tables 5 and 6). Significantly gender differences were found in the proportion of meat consumed in the control period: men (*M* = 211.0 g, SD = 46.77) consumed more meat than women (*M* = 199.8 g, SD = 47.61; *t*(1, 527) = 2.71, *p* < .01). In addition, during the intervention period, we found that participants with children ate significantly more vegetables from their plates (*M* = 115.7 g, SD = 40.97) and overall (*M* = 177.2 g, SD = 59.09) than participants who had no children (*M* = 106.7 g, SD = 44.99; *t*(1, 459) = 2.09, *p* < .05 and *M* = 161.2 g, SD = 59.12; *t*(1, 459) = 2.66, *p* < .01, respectively). Additionally, positive correlations between age and the amount of vegetables consumed were found in both the control period and the intervention period (*p* < .01), implying that older people ate more vegetables.

**Table 5:** Sub-group analyses for control period and intervention period

Categorical variables: means and standard deviations are reported	Vegetable consumption in grams (plate)		Vegetable consumption in grams (total, incl. side dishes)		Meat/ fish consumption in grams	
	Control period	Intervention period	Control period	Intervention period	Control period	Intervention period
Gender						
Male	64.1 (19.4)^a^	116.1 (41.7)^a^	134.6 (45.8)^a^	175.5 (60.7)^a^	211.0 (46.8)^a^	183.1 (44.3)^a^
Female	62.2 (20.8)^a^	110.1 (43.0)^a^	133.1 (45.1)^a^	169.7 (58.4)^a^	199.8 (47.6)^b^	183.0 (43.2)^a^
Children						
Yes	64.0 (19.3)^a^	115.7 (41.0)^a^	136.1 (45.8)^a^	177.2 (59.1)^a^	204.7 (47.6)^a^	183.7 (43.4)^a^
No	61.1 (21.5)^a^	106.7 (45.0)^b^	128.6 (44.3)^a^	161.2 (59.1)^b^	206.9 (47.6)^a^	184.3 (44.5)^a^
Reason of visit						
Business	68.8 (11.1)^a^	133.8 (37.7)^a^	148.4 (38.2)^a^	210.4 (44.0)^a^	195.8 (29.0)^a^	182.7 (45.0)^a^
Leisure	63.0 (20.2)^a^	112.3 (42.4)^a^	133.7 (45.5)^a^	171.2 (59.3)^b^	205.4 (47.8)^a^	183.0 (43.4)^a^

Standard deviations are reported in brackets. Means with a different superscript ^a, b^ indicate a significant difference (*p* < .05)

**Table 6:** Sub-group analyses for control period and intervention period

Continuous variables: Pearson correlation-coefficients (r) are reported	Vegetable consumption in grams (plate)		Vegetable consumption in grams (total, incl. side dishes)		Meat/ fish consumption in grams	
	Control period	Intervention period	Control period	Intervention period	Control period	Intervention period
Frequency of visit	−.09*	−.06	−.13**	−.11*	.00	.02
Frequency of dining	−.03	.07	.07	.07	.07	.04
Age	.09	.26***	.13**	.24***	−.02	.08
Importance of vegetables when dining	.21***	.29***	.29***	.29***	−.02	.05
I highly value a tasteful meal.	−.03	−.04	−.02	−.03	−.07	.00
I am very involved with my food choices	−.00	.10*	.06	.13**	−.07	−.03
I have been eating vegetables since I was a child.	.01	.04	.03	.03	−.01	.07
I am very particular about the vegetables I will eat.	.03	−.06	−.02	−.09	−.11*	−.12*
I like vegetables	.10*	.13**	.14**	.16***	−.03	.08
I would like to eat more vegetables	.07	.03	.15***	.05	−.06	−.01
I know how much vegetables I have to eat during the day.	.02	.10*	−.02	.11*	−.07	.02
I find it difficult to assess whether I eat enough vegetables.	−.01	−.09	.05	−.10*	−.05	.01

*** *p* < .001; **p < .01; *p < .05

Furthermore, in the intervention period, participants who visited the restaurant for business consumed a significantly higher total amount of vegetables (*M* = 210.4 g, SD = 43.99) than participants who visited the restaurant for leisure (*M* = 171.2 g, SD = 59.35; *t*(1, 467) = 2.54, *p* < .05). Significantly correlations were found between the frequency of visits and the total amount of vegetables consumed in the control and intervention period (*p* < .05) as well as between the frequency of visits and vegetable consumption from plates in the control period (*p* < .05). These correlations implied that participants who visited the restaurant more frequently tended to eat less vegetables.

Finally, strong positive correlations were found between the reported importance of eating vegetables when dining out and the amount of vegetables consumed from plates as well as total vegetable consumption in both the control period and the intervention period (*p* < .001); between involvement in food choices and the amount of vegetables consumed from plates as well as total vegetable consumption in the intervention period (*p* < .05); between liking of vegetables and the amount of vegetables consumed from plates and overall for both the control period and the intervention period (*p* < .05); and between willingness to eat more vegetables and total vegetable consumption during the control period (*p* < .001).^2^ Therefore, consumers who already had a positive attitude towards vegetables were more likely to ate more vegetables. In addition, knowledge of the amount of vegetables one has to eat during the day was positively correlated with the amount of vegetables consumed from plates and total vegetable consumption in the intervention period (*p* < .05); in contrast, difficulty assessing whether one has eaten enough vegetables was negatively correlated with the total amount of vegetables consumed for the intervention period (*p* < .05). Finally, fastidiousness with regard to vegetables was somewhat strikingly negatively correlated with meat consumption during the control period as well as the intervention period (*p* < .05).

## Discussion

### Discussion of main results

The aim of this research was to investigate whether increased portion sizes of vegetables and decreased portion sizes of meat in main dishes increased the amount of vegetables consumed in a real-life restaurant setting without affecting customer’s satisfaction. The results of this real-life experiment showed that during the intervention period, participants consumed 87% more vegetables from their plates compared to the control period. Taking into account that fewer vegetables were eaten from side dishes during the intervention period, participants still consumed 30% more vegetables in total (from the plates and the side dishes) during the intervention period than during the control period. These findings are in line with Rolls et al., who found that increasing the portion size of healthy food products with a low energy density led to a higher consumption of these healthy food products [26].

Although participants evaluated the amount of vegetables to be higher and the amount of meat lower in the intervention period compared to the control period, they remained (very) satisfied with their main dish. Satisfaction with the main dish was only slightly lower during the intervention period than during the control period. In addition, satisfaction with the visit to the restaurant did not differ, and no complaints were received during the intervention period. These results suggest that portion size could significantly affect how much food is consumed while largely not affecting satisfaction. These findings seem to be consistent with another field study by Diliberti et al., in which no differences in satisfaction between restaurant meals with a higher and lower portion size were found [18].

### Discussion of other results

The results of the current study showed a significant interaction between the intervention and the location of the restaurants. This meant that the location of the restaurant was an important moderator of the results and that the intervention seemed to work better in some restaurants than others. More specifically, the intervention effect seemed to be largest in location 1 compared to locations 2 and 3. In additional analyses, we investigated whether we could identify explanatory factors for this difference in the demographic, lifestyle and nutrition-related characteristics included in this study. We did not find differences in gender, age or education per location nor in any of the lifestyle characteristics. There were two significant differences in the nutrition-related characteristics. Visitors at location 1 reported visiting restaurants more often than visitors at locations 2 and 3. Furthermore, visitors at location 1 (regardless of intervention or control condition) perceived the amount of vegetables served to be lower than the perceived amount by visitors at locations 2 and 3. We can only speculate regarding the reasons for these differences. It could be that there was a difference in accuracy with regard to the measurements or servings of the plates and that the portions were smaller or larger in practice than we intended.

The importance of eating vegetables when dining out was different during the intervention and control period. This finding was surprising because we did not expect to observe any significant differences between the control and intervention period for this measure. However, because the study was conducted in a real-life setting, it was not possible to completely randomize participants between an intervention and control group. Moreover, other variables that were not measured that were beyond control of the experiment (e.g., the weather, temperature in the restaurant, information campaigns about eating vegetables) could have affected the importance of eating vegetables during the intervention and control period. Finally, it is also possible that the intervention itself (i.e., the amount of vegetables served) influenced the importance of vegetable intake. For example, serving a larger portion could be related to a lower importance of eating vegetables and vice versa. Future research should help clarify whether this was indeed the case.

### Theoretical and practical implications

This study has several strengths that improve our understanding of what role restaurants could play in offering healthier food choices (i.e., more vegetables, less meat). First, we showed that the effects of increased portion sizes on consumption behavior identified in previous studies remained true for increased portion sizes of healthy food. Second, we conducted a field experiment in a real-life restaurant setting. To the best of our knowledge, this is one of the first studies to show that modifying portion sizes in a restaurant setting could lead to an increase in consumption of healthy foods. Most studies have been conducted in a lab setting. This is an especially interesting finding given that unhealthy products are generally preferred when hedonic goals are salient (which is the case in restaurants) [27]. Unknowingly increasing the portions of vegetables on plates could thus circumvent these hedonic goals. Finally, we used different data sources to confirm the robustness of the results: observational data on vegetable consumption and self-reported data on customer satisfaction and experiences. By complementing self-reported measures with more objective measures, we were more able to control for the fact that consumers have difficulty assessing their own consumption behavior. In addition, by measuring net plate weights, we provided a novel method of measuring dietary intake and dietary behavior.

From a practical perspective, the results of this study imply that modifications of portion sizes in restaurants are an effective tool for stimulating vegetable consumption and lowering meat consumption. By using this tool, restaurants could play an important role in mitigating the increase in overweight and obesity and could improve diet quality by offering healthier and more sustainable menus. Moreover, serving more vegetables and less meat in restaurants will contribute to having a lower environmental impact, as the production and processing of vegetables requires less water and has less CO_2_ emissions than the production of meat. A Dutch research report from 2013 showed that changing the composition of plates in restaurants by decreasing the portion size of meat and fish by 10% and increasing the vegetable portion size by 22.1% lowered the ecological footprint by 5.4%, the carbon footprint (CO_2_) by 6.4% and the water footprint by 6.3% [28].

### Limitations and future research

This study has several limitations that could be addressed in future research. First, we did not measure the consumption of appetizers and desserts in our study, since these courses were not weighed. The consumption of main dishes could have been affected by other courses that the participants consumed. In addition, substitution effects or other side effects might have occurred. In this study, people ate more vegetables and less meat, but they could have increased their consumption of unhealthy foods in their dessert orders. As the current study did not measure the consumption of other courses, we could not establish to what extent these effects occurred. It would be interesting for future research to examine whether concepts such as ego depletion (i.e., depleted willpower by a task that requires a lot of self-control) play a role in side effects that occur as a result of modifying vegetable portion sizes in restaurants [29].

Similarly, we did not measure beverage consumption, which could have affected the amount of food that the participants ate. In this respect, previous studies found that alcohol consumed before or with meals tended to increase appetite and food intake [30, 31]. In addition, the consumption of alcoholic beverages could lead to a greater need for salty and fatty foods [31]. Future research should account for all the different courses and drinks consumed to obtain a more complete picture of the conditions under which portion size modifications have the greatest impact.

Finally, in our study, we did not measure every component of the main dishes and side dishes before they were served. Instead, for feasibility reasons, the averages of each component were calculated each evening and were used as the average serving portion for that specific main dish or side dish for that specific evening. Future research could improve the accuracy of these measures by weighing all individual dishes before consumption.

## Conclusions

In conclusion, this is one of the first studies to investigate the effects of modifying the portion sizes of vegetables and meat included in a main dish in a real-life restaurant setting. We found that increased portion sizes of vegetables in combination with decreased portion sizes of meat increased vegetable consumption while simultaneously enabling guests to remain (very) satisfied with their restaurant visit. These findings provide support for the idea that modifications of portion sizes are an effective tool for enhancing healthy and sustainable diets in restaurants.

## Footnotes

1 For readability, we only discuss decreased portion sizes of meat throughout the article, but some fish menus were also included in the study.

2 Notice that we conducted a factor analysis to check whether one or more composite measures could be extracted based on these items. The results of the factor analysis showed no interrelationships among items that warranted extracting a sensible number of factors. Therefore, we decided to treat these items separately.
